# A commercial scale evaluation of the effects of post-weaning management system of dairy × beef hybrid steers compared to native beef steers on performance, carcass characteristics, and net returns

**DOI:** 10.1093/tas/txaf118

**Published:** 2025-08-30

**Authors:** Ally Grote, Tom Fanning, Eric A DeVuyst, Zane Grigsby, Justin Crosswhite, Paul Beck

**Affiliations:** Oklahoma State University Department of Animal and Food Sciences, Stillwater, OK, 74078USA; Pratt Feeders LLC, Pratt, KS, 67124USA; Oklahoma State University Department of Agricultural Economics, Stillwater, OK, 74078USA; Oklahoma State University Department of Animal and Food Sciences, Stillwater, OK, 74078USA; Oklahoma State University Department of Animal and Food Sciences, Stillwater, OK, 74078USA; Oklahoma State University Department of Animal and Food Sciences, Stillwater, OK, 74078USA

**Keywords:** Dairy, Beef, Economics, Performance, Liver Abscesses

## Abstract

Our objective was to determine the effect of calf-fed (**CF**) or yearling-fed (**YF**) finishing systems on performance and carcass characteristics of beef × dairy (**DB**) crossbred steers compared to native beef (**NB**) steers. The NB steers (*n* = 160) were acquired from Capitol Land and Livestock in Schwertner, Texas. The DB steers (*n* = 184) were acquired from 5-Star Dairy in Hart, Texas. The CF (*n* = 194) steers were transported directly to a commercial feedyard (Buffalo Feeders, Buffalo, OK) from the source. Steers in the YF system (*n* = 150) were transported to the Marvin Klemme Research Range, near Bessie, OK, to graze mixed grass prairie for 144-d before finishing. At Buffalo Feeders, steers were sorted by finishing system and breed-type into commercial size pens, so each breed-type  ×  system combination were in a single pen. Data were analyzed using SAS 9.4 Mixed Procedure (SAS Institute, Cary, NC) with individual steer as the experimental unit. For CF, initial finishing BW did not differ for DB and NB (*P *= 0.11), while the initial finishing BW of NBYF was greater (*P *< 0.01) than DBYF due to NBYF steers having greater ADG on pasture. At reimplant, BW of YF steers was greater (*P *< 0.01) than CF steers. The DBYF steers had the greatest overall ADG (*P *< 0.01) with NBCF having the least ADG (*P *< 0.01) with NBYF and DBCF being intermediate. Back-fat thickness (*P *= 0.03) was greatest in NBCF, and DBYF having the least BFT, while DBCF and NBYF were intermediate. Overall liver abscesses had breed-type (*P *< 0.01) and finishing system effects (*P *= 0.01) with DB and YF steers having higher incidence of liver abscesses. Total net return was greatest (*P *< 0.01) in NBYF followed by DBYF, DBCF, and NBCF, respectively. Grazing beef × dairy steers before feedlot finishing can improve animal performance and certain carcass characteristics, but there is still a high incidence of liver abscesses. Since DB systems had intermediate returns, these animals can be competitive to their NB counterparts, but the higher prevalence of liver abscesses needs to be further researched.

## Teaser

Grazing before feedlot entry boosts performance and profitability of dairy × beef steers, but elevated liver abscess rates present a health management challenge.

## INTRODUCTION

Male dairy calves are typically seen as a byproduct of the dairy industry (Jaborek et al., 2023), but dairy cows in general produce 3 to 4 million dairy calves for the feedlots each year (Drouillard, 2018) -- a substantial contribution to the United State beef supply. Due to the 2012 drought and subsequent recession of beef cow numbers, there was a significant increase in finishing of dairy-type calves from 2011 to 2016, helping maintain beef supply (Boykin et al., 2016). When native beef supply began to return to normal in 2016, the Holstein bull calf lost nearly all value due to reduced market opportunities for both surplus females and male calves (Basiel and Felix, 2022). Dairy farmers began looking for other avenues for these calves and began mating a portion of their genetically inferior females to beef breed bulls to not only start new lactations but add value to surplus calves in the dairy industry (Basiel and Felix, 2022). These resulting dairy × beef calves are currently more valuable to buyers than a straight-bred dairy bull calf (Wilson et al., 2020; Basiel and Felix, 2022) and closer in value to native beef breeds (McCabe et al., 2022) due to improved performance and carcass characteristics (Basiel and Felix, 2022). But, there is limited modern data on growth and performance of dairy × beef crossbred calves reared in the United States beef industry (Basiel and Felix, 2022).

Calf-fed versus yearling-fed management practices look different for dairy-type and beef-type cattle even though similar terminology is used. Calf-fed systems in the beef industry are when calves are placed on high-concentrate diets directly after weaning at approximately 28 wks of age (Reuter and Beck, 2013). Typically, a calf-fed system in the dairy industry is typically defined as calves weaned at eight weeks of age and are entering the feedlot at 12 – 16 wks of age weighing 125 to 182 kg and placed on an energy dense calf starter (Jaborek et al., 2023). In the beef industry, yearling-fed systems are an essential part of beef production and typically consist of stocker calves that are placed on pastures or grown on diets containing high-quality forages for a length of time before entering the feedlot at 12 to 16 mo of age (Reuter and Beck, 2013).

When dairy × beef crossbred calves are finished these calves are not only more efficient but also spend fewer days on feed and have a greater ADG than purebred dairy calves (Basiel and Felix, 2022). However, there is limited data on growth and performance of these crossbred calves reared in the United States compared to their native beef counterparts, especially when compared to the literature on calf-fed dairy calves (Basiel and Felix, 2022); and there is limited current scientific data comparing native beef calves and dairy × beef crossbred calves reared in extensive pasture based management strategies. Therefore, the objective of the study was to determine animal performance, efficiency, carcass quality, and economic returns of beef × dairy crossbred steers (**DB**) and native beef steers (**NB**) placed in either a calf-fed (**CF**) or yearling-fed (**YF**) finishing system.

## MATERIALS AND METHODS

All procedures were approved by the Institutional Animal Care and Use Committee at Oklahoma State University (Animal Care and Use Protocol Number: IACUC-22-16)

### Calf-Fed System

Beef × dairy (DB; *n* = 109) were acquired from 5-Star Dairy in Hart, Texas, and the native beef (NB; *n* = 85) were acquired from Capitol Land and Livestock in Schwertner, Texas. All steers were transported to a commercial feedlot (Buffalo Feeders in Buffalo, OK). Upon arrival at the feedlot, initial BW was recorded and steers were dewormed and vaccinated for infectious bovine rhinotracheitis and bovine virus diarrhea (Pyramid 3 LPH + Presponse SQ Boehringer Ingelheim Animal Health, Ingelheim am Rhein, Germany), and implanted with 20 mg of estradiol benzoate, 200 mg of progesterone, and 29 mg of tylosin tartrate (Component E-S with Tylan, Elanco US Inc., Greenfield, IN). Due to constraints of pen availability, steers were assigned to a single commercial-scale finishing pen for each breed-type and finishing system.

The diets fed in the feedlot are displayed in Table 1. The high roughage growing diet was Diet 1 and step-up diets were Diets 2, 3, and 4. Diet 5 was the finishing diet. The NBCF steers were fed the growing diet (Diet 1) for 97 d supplying 26% roughage, 15% CP and 1.2 Mcal NEg/kg (DM basis). After the growing diet, NB steers were transitioned through step-up Diets 2 and 3 for 5 d each with a 3-d transition period between diets which blended the current diet with the next diet. Native beef steers were transitioned to the finishing diet (Diet 5) on d 111. The DBCF were fed each of the successive step-up diets for 19 d each with a 3-d transition period. After the step-up diets on d 64, DBCF were transitioned to the finishing diet and fed until shipping for slaughter.

**Table 1.** Ingredients and chemical composition of finishing diets fed in the feedlot.

	Diet^1^				
Item	1	2	3	4	5
**Ingredient Composition, % as-fed**					
Alfalfa Blend	25.8	35.0	26.0	16.0	7.0
Flaked Corn	32.0	34.0	44.0	55.0	61.8
Wet Distillers Grains	30.0	24.0	21.0	18.0	15.0
Corn Syrup	7.5	3.0	4.0	5.0	5.0
Breading	-	-	-	-	4.0
Fat	-	-	0.5	1.0	1.3
Starter Liquid	-	3.0	2.0	1.0	
Finisher Liquid	3.8	-	1.5	3.0	5.0
Micros	1.0	1.0	1.0	1.0	1.0
**Chemical Composition** ^2^					
DM, %	63.8	68.0	68.4	68.8	70.2
CP, %	15.0	13.0	13.0	12.9	13.0
Fat, %	3.6	3.4	4.1	4.8	7.8
NEm, Mcal/kg DM	1.8	1.7	1.9	2.1	2.4
NEg, Mcal/kg DM	1.2	1.1	1.3	1.4	1.6

^1^Diet 1 = growing diet fed to NBCF for 97 d; Diet 2, 3, and 4 = step-up diets fed to NBCF for 5 d each with a 3-d transition period between them and fed to DBCF for 19 d each with a 3-d transition period; DBYF were fed Diet 2 and 3 for 9 d each with 3-d transition period and fed Diet 4 for 4 d with 3-d transition; NBYF were fed Diet 2 for 10 d, Diet 3 for 5 d, and Diet 4 for 3 d with a 3-d transition period between each diet; Diet 5 = finishing diet fed after step-up diets at d 111, d 64, d 29, and d 25 for NBCF, DBCF, DBYF, and NBYF, respectively, until slaughter.

^2^Analysis conducted using near-infrared spectroscopy by ServiTech Inc. (Dodge City, KS).

Native beef steers were reimplanted on d 78, and DB steers were reimplanted on d 108. All steers were reimplanted with either 200 mg of trenbolone acetate and 28 mg of estradiol benzoate (Synovex One Feedlot, Zoetis Animal Health) or 200 mg of trenbolone acetate and 28 mg of estradiol benzoate (Synovex Plus, Zoetis Animal Health). At reimplant, individual animal BW were recorded, and ultrasounds were performed by trained personnel using an Aloka 500V system equipped with a 17-cm 3.5-MHz transducer probe (Aloka Co. Ltd., Wallingford, CT) for the measurement of ribeye area (REA) and backfat thickness (BFT). Steers were placed into heavy and light sorts based on reimplant weight. Calf-fed NB steers were shipped for slaughter on 20 February 2024 and 02 April 2024, and DBCF steers were shipped for slaughter on 23 January 2024 and 20 February 2024. Steer BW at slaughter were based on carcass adjusted BW, calculated as hot carcass weight (**HCW**) divided by the pen average dressing percentage divided by 100.

### Yearling-Fed System

Beef × dairy (DB; *n* = 75) were acquired from 5-Star Dairy in Hart, Texas, and the native beef (NB; *n* = 75) were acquired from Capitol Land and Livestock in Schwertner, Texas. All steers were transported to the OSU Marvin Klemme Research Range Station (**MKRRS**), near Bessie, Oklahoma (35°25’00.4” N, 99°03’42.6” W). Upon arrival to MKRRS, steers were placed into a holding pen with ad libitum access to mixed grass hay and water for approximately 48 h. All steers were weighed at 24 h and 48 h post-arrival to MKRRS. Steers were allocated by BW and breed-type into 3 groups, which were randomly assigned to one of three pastures to achieve a stocking rate of 2.43 ha per animal (pasture 1; n = 39, pasture 2; n = 52, pasture 3; n = 59). The stocking rate at this site was based on data from Beck et al. (2020). Pastures were also balanced for breed type (pasture 1: DB = 21, NB = 18, pasture 2: DB = 26, NB = 26, pasture 3: DB = 28, NB = 31). Steers were supplemented with extruded 100% DDGS cubes (MasterHand Milling, Lexington, NE) at 0.45 kg per steer per day for the first 53 d. Steers were supplemented with 1.13 kg per steer per day from day 54 to end of trial to meet forage nutrient deficiencies due to increasing forage maturity. All steers had ad libitum access to stocker mineral (MasterHand Milling, Lexington, NE) during the grazing period.

The MKRRS is characterized by rolling Red Shale uplands (2 – 15% slopes) dissected by deep drainages with Cordell silty clay loam soils, which are shallow (25 to 36 cm) and contain numerous rocky outcrops of hard red siltstone. These Red Shale sites support mixed-grass prairie as the potential climax natural vegetation (Gillen et al., 2000). Species composition was previously published by Gillen et al. (2000) and the major grass species identified on this site in 1990 were sideoats grama (*Bouteloua curtipendula* (Michx.) Torr.) other short grass native species including buffalograss (*Buchlöe dactyloides* (Nutt.) Englem.), with lesser amounts of blue grama [*B. gracilis* (Willd. ex Kuth) Lag. Ex Griffiths] and hairy grama [*B. hirsuta* Lag.]; silver bluestem [*Bothriochloa saccharoides* (Sw.) Rydb.]; red threeawn [*A. purpurea* Nutt.]; and minor amounts of tallgrass prairie species, primarily little bluestem [*Schizachyrium scoparium* (Michx.) Nash.]. The major forbs (identified by Gillen et al. (2000) included: western ragweed [*Ambrosia psilostachya* DC.] and curlycup gumweed [*Grindelia squarrosa* (Pursh.) Dun.]. Broom snakeweed [*Gutierrezia sarothrae* (Pursh) Britt. & Rusby], a poisonous plant, was found in scattered areas (Gillen et al., 2000). Standing forage heights were collected each week in 20 locations throughout each pasture and available forage determined based on 75 to 90% ground cover and 20-cm residue height using estimates provided by Rocateli (2017) for mixed native grass pastures. Forage grab samples were collected at that time from the area around each forage height determination. Forage samples were dried in a forced air oven at 50 °C and ground to pass through a 1mm screen (Fritsch, Pulverisette 19, Pittsboro, NC) and composited within pasture for each sampling date. Following grinding, the chemical composition of the whole plant samples were estimated via near infrared reflectance spectroscopy (NIRSD2500 F, Foss Analytics, Eden Prairie, MN).

For weigh days at MKRRS, steers were gathered a day prior and held together in a dry lot. Steers received ad libtum access to mixed grass hay and water overnight. Steers were weighed on d 53, 144, and 145. Steer body composition was estimated via ultrasound measurements taken by a trained professional using an Aloka 500V system equipped with a 17-cm 3.5-MHz transducer probe (Aloka Co. Ltd., Wallingford, CT) on all steers on d 145 prior to transport for finishing.

After the grazing period, animals were transported approximately 204 km to a commercial feedlot (Buffalo Feeders, Buffalo, OK), to be followed through the finishing phase and evaluate finishing system and breed type effects on feedlot animal performance and carcass characteristics. Upon arrival at the commercial feedlot, initial BW was recorded and all YF steers were dewormed and vaccinated for infectious bovine rhinotracheitis and bovine virus diarrhea (Pyramid 3 + Presponse, Boehringer Ingelheim Animal Health, Ingelheim am Rhein, Germany), and implanted with 100 mg of trenbolone acetate and 14 mg of estradiol benzoate (Synovex Choice, Zoetis Animal Health, Parsipanny, NJ). Due to constraints of pen availability, steers were assigned to a single commercial-scale finishing pen for each breed-type and finishing system.

The previously described diets fed in the feedlot are displayed in Table 1. Yearling-fed DB steers were fed step-up Diets 2 and 3 for 9 d each, with a 3-d transition period. Diet 4 was fed to DBYF for 4 d before transitioning to the finishing diet at d 29. Native beef steers were fed step-up Diet 2 for 10 d, Diet 3 for 5 d, and Diet 4 for 3 d with a 3-d transition period between each step-up diet. After the step-up diets, NBYF steers were transitioned to the finishing diet at d 25 and fed through shipping for slaughter.

All steers were reimplanted on d 87 with either 200 mg of trenbolone acetate and 28 mg of estradiol benzoate (Synovex One Feedlot, Zoetis Animal Health) or 200 mg of trenbolone acetate and 28 mg of estradiol benzoate (Synovex Plus, Zoetis Animal Health). At reimplant, individual animal BW were recorded, and ultrasounds were performed by trained personnel using an Aloka 500V system equipped with a 17-cm 3.5-MHz transducer probe (Aloka Co. Ltd., Wallingford, CT) for the measurement of ribeye area (REA) and backfat thickness (BFT). Steers were placed into heavy and light sorts based on reimplant weight. Native beef YF steers were shipped for slaughter on 23 April 2024 and 07 May 2024, and DBYF steers were shipped for slaughter on 30 April 2024 and 07 May 2024. Steer BW at slaughter were based on carcass adjusted BW, calculated as HCW divided by the pen average divided by 100.

### Carcass Characteristics

Upon being deemed finished by the manager at the commercial feedyard at the end of the finishing phase, animals were transported 142 km to a commercial abattoir for slaughter (National Beef Packing Co. LLC, Liberal, KS). At slaughter, HCW, USDA Quality Grade (**QG**) and yield grade (**YG**), marbling score (200 = Standard+, 300 = Select+, 400 = Choice−, 500 = Choice°, 600 = Choice+, 700 = Prime−, 800 = Prime°, 900 = Prime+; USDA-AMS, 2017), REA, and BFT were collected by trained personnel from the abattoir via carcass camera imaging, and HCW as a percentage of BW was considered the average hot yield for each slaughter group. Liver abscess scores were manually scored and recorded according to the Elanco Liver Check Scoring System: A− = 1 or 2 small abscesses or inactive scars; A = 2 to 4 small active abscesses; A+ = multiple small abscesses or 1 or more large active abscesses; A+AD = liver adhered to part of the gastrointestinal tract or diaphragm or both; A+OP = ruptured abscesses; A+AD/OP = liver adhered to part of the gastrointestinal tract or diaphragm or both and ruptured abscess.

### Modeled Shrunk and Empty Body Composition, Feed Intake, and Feed Efficiency

Since steers were fed in commercial feedlot pens, equations, developed by Guiroy et al. (2001), and Tedeschi et al. (2004), were used to parse DMI within each pen to individuals in order to estimate daily individual DMI, total individual DMI, and feed efficiency (**G:F**) as described by Adams et al. (2023). These equations (Guiroy et al., 2001; and Tedeschi et al., 2004) were used to estimate composition of gain including: adjusted final shrunk BW (**AFSBW**), shrunk weight gain (**SWG**), empty body weight (**EBW**), empty body weight gain (**EWG**), empty body composition of fat (**EBF**), and DOF required to reach 28% EBF during the finishing period. These estimates were then used along with actual average pen level DMI for determining an estimate of individual feed DMI as described by Adams et al. (2023).

### System Net Returns

Purchase prices and feed costs were based on a weighted average to account for seasonal differences throughout the year (Table 2). Costs of gain during the finishing period were calculated as total feed costs per animal in the feedyard divided by total gain per animal in the feedyard. The total system costs of gain were calculated as the total grazing costs for YF steers and total feed costs in the feedyard divided by the total gain from the stocker and finishing periods. In addition, total system net returns were calculated as carcass value minus total system costs, including the initial cost of the calf, total cost of gain (**COG**) for both grazing and feedlot, the opportunity costs of investment, feed, yardage, and veterinary expenses using an interest rate of 7% per year, and actual veterinary care and medicinal costs. Yardage was included in the feed cost for the finishing period, and the feed cost was $0.40 per kg on a weighted average. For the grazing portion, supplement cost was calculated using a price of $0.50 per kg of feed, and the grass cost was calculated using a price of $1.54 per kg of gain.

**Table 2.** Costs and prices received for each system for the calculation used for the weighted average values used in the analysis of net returns.

System^1^	Purchase Price/kg	Feed Price, $/kg	Carcass Base Grid Price/kg	Carcass Premiums and Discounts					
				Choice +	Prime	YG1	YG2	YG5	CAB^2^
NBCF-HS	5.41	0.42	6.28	+10.03	+13.32	+5.00	+3.00	-20.00	+3.63
NBCF-LS	5.41	0.42	6.44	+9.49	+12.69	+4.00	+2.00	-20.00	+3.00
NBYF-HS	3.49	0.37	6.31	+5.21	+12.60	+4.00	+2.00	-20.00	+3.00
NBYF-LS	3.49	0.37	6.39	+5.26	+13.59	+4.00	+2.00	-20.00	+3.00
DBCF-HS	4.90	0.43	6.01	+18.58	+20.79	+5.00	+3.00	-20.00	+3.00
DBCF-LS	4.90	0.43	6.28	+10.03	+14.90	+4.00	+2.00	-10.00	+3.00
DBYF-HS	4.39	0.37	6.32	+4.68	+14.11	+4.00	+2.00	-10.00	+3.00
DBYF-LS	4.39	0.37	6.39	+5.26	+15.72	+4.00	+2.00	-10.00	+3.00
Weighted Average	4.59	0.40	6.32	+8.13	+14.77	+4.23	+2.23	-16.34	+3.09

^1^Base prices, premiums, and discounts for native beef (NB) or dairy-beef (DB) steers in yearling-fed (YF) or calf-fed (CF) systems with heavy sorts (HS) and light sorts (LS). .

^2^CAB = Certified Angus Beef.

Carcass values were calculated using a weighted average of the actual base grid prices used to account for changes in cattle prices between slaughter dates (Table 2). The weighted average base grid price used to calculate carcass value was a base price of $6.32/kg, with QG adjustments of $14.77 and $8.13 for Prime and Choice, respectively; YG adjustments of $4.23 (YG = 1), $2.23 (YG = 2), $0.00 (YG = 3), −$10.00 (YG = 4), and −$16.34 (YG = 5); weight adjustments of −$30.00 (≤ 261 kg HCW) and −$15.00 (≥ 500 kg HCW); $3.09, $0.50, $2.00 premiums for Certified Angus Beef, Black Canyon Premium Reserve, and Certified Hereford Beef, respectively; and discounts of −$10.00 for no roll, −$25.00 for hard bone, and −$12.00 for over 30 mos of age

### Statistical Analysis

All statistical analyses were performed using SAS software, version 9.4 (SAS Institute Inc., Cary, NC). Animal performance and carcass characteristics were analyzed as a Completely Randomized Design using the Mixed procedure. Carcass USDA QG and incidence of liver abscesses were analyzed using the Glimmix procedure. For all variables, breed type (NB vs DB), finishing system (YF vs CF), and their interaction were fixed effects. The Satterthwaite denominator degrees of freedom approximation option was used in the model statement to request that the denominator degrees of freedom in t-tests and F-tests be computed according to a general Satterthwaite approximation. Individual animal was treated as the experimental unit within each production system. The LSMEANS statement was included in the model to determine the least squares means, the predicted differences option in SAS was used to separate the effects of treatment, and the largest standard error is reported. Differences were declared significant at *P* ≤ 0.05, and tendencies declared at 0.10 ≥ *P* > 0.05.

## RESULTS AND DISCUSSION

### Animal Performance at MKRRS

The average forage availability was 1,146 ± 403.5 kg/ha (DM basis) throughout the summer grazing season. Forage DM, CP, and 48-hr in vitro true DM disappearance were 69.2 ± 4.8%, 4.8 ± 1.13%, and 68.0 ± 2.66%, respectively. Based on NASEM (2016) estimations, CP would be considered deficient at all sampling dates for all classes of beef cattle, while the IVDMD would be sufficient to promote positive growth rates of grazing steers. The low protein content in relation to the potentially digestible energy of the forage created an unbalanced IVDMD:CP ratio (Moore et al., 1999) and indicates a ruminal N deficiency. Correction of this ruminal N deficiency with supplementation would likely result in positive associative effects with adequate forage availability (Moore et al., 1999). The forage availability and nutritive content were similar to other estimates at this research site.

The effect of breed type on grazing performance at the MKRRS is presented in Table 3. Initial BW for grazing was 8 ± 3 kg greater (*P* = 0.01) in DB steers, but at d 53 of grazing, NB steers had 16 ± 4 kg greater (*P* < 0.01) BW than DB steers. Average daily gain was 0.44 ± 0.04 kg/d less (*P* < 0.01) in DB calves than NB for the first part of the grazing season. During the late summer, ADG was 0.08 ± 0.02 kg/d greater (*P* < 0.01) in NB calves than DB. At the end of the grazing season, NB steers weighed 24 ± 5 kg more (*P* < 0.01) and had an overall greater (*P* = 0.01) ADG than DB steers, where NB steers ADG was 0.77 kg/d and DB steers ADG was 0.55 kg/d. The lower ADG for the first 53 d in DB calves in the current study is likely due to differences in previous diet before arrival at MKRRS for grazing. There was also likely a period of adaptation to grazing in the unfamiliar open-range environment (Provenza, 1995), especially since ADG was closer to that of NB calves from d 53 to d 144. However, grazing ADG was less than previous observations with growing NB steers provided supplement throughout the grazing season at the MKRRS and in the range of performance expected of unsupplemented growing steers at the research site (Grigsby et al., 2023).

**Table 3.** Grazing performance of the yearling fed beef × dairy crossbred and steers native beef steers at the OSU Marvin Klemme Range Research Station.

	Treatments^1^			
Item^2^	NB	DB	SEM^3^	*P*-value
BW, kg				
Initial	267	275	3.0	0.01
d 53	301	285	3.9	<0.01
Final	379	356	4.7	<0.01
ADG, kg·hd^-1^				
d 0 – 53	0.64	0.20	0.042	<0.01
d 53 – 144	0.86	0.78	0.022	<0.01
Overall	0.77	0.55	0.023	<0.01

^1^Treatments consisted of grazing beef × dairy (DB) and native beef (NB) steers on native rangeland for 144 d before finishing.

^2^BW = body weight, and ADG = average daily gain.

^3^Standard error of the mean. .

### Animal Performance in the Finishing Period

Bodyweight and gain performance of steers by breed type and finishing system are presented in Table 4. Initial feedlot and reimplant BW were affected by a breed type × finish interaction (*P* ≤ 0.01). At feedlot entry, BW of NBYF was greatest (*P* ≤ 0.01) compared with other breed type × finishing system combinations. The DBYF system had greater (*P* ≤ 0.01) initial finishing BW than either CF systems, and the CF systems did not differ (*P* = 0.11) from each other for initial feedlot BW. However, at reimplant, both YF systems had a greater (*P* ≤ 0.01) BW than both CF systems but reimplant weight for the YF systems did not differ (*P* = 0.73) between breed types. The DBCF system weighed the least (*P* ≤ 0.01) at reimplant. While there was no interaction at final harvest BW (*P* = 0.47), there were breed type and finishing system effects at final harvest BW. Beef × dairy calves exhibited greater (*P* ≤ 0.01) final BW than NB calves, and the YF system had a greater (*P* ≤ 0.01) final BW than the CF system. For DOF, there was no interaction (*P* = 0.16) between breed type and finishing system, but NB calves were on feed for 4 ± 1 fewer (*P* = 0.01) d than DB calves, and those calves in the YF system were on feed for 98 ± 2 d less than (*P* ≤ 0.01) CF calves. There was no effect of breed type, finishing system or their interaction (*P* ≥ 0.21) on mortality rate.

**Table 4.** Effect of calf-fed or yearling-fed finishing systems on performance in beef × dairy crossbred steers compared to native beef steers.

	Treatments^1^					Effect *P-*values		
Item^2^	NBCF	DBCF	NBYF	DBYF	SEM^3^	System	Breed-type	System × Breed-type
BW, kg								
Initial	268^c^	262^c^	359^a^	338^b^	3.9	<0.01	<0.01	0.01
Reimplant	452^b^	418^c^	516^a^	519^a^	6.3	<0.01	<0.01	<0.01
Slaughter	665	710	691	727	8.8	<0.01	<0.01	0.47
DOF	296	302	201	202	2.0	<0.01	0.01	0.16
Mortality, %	8.2	13.8	2.7	2.7	0.04	<0.01	0.34	0.34
ADG, kg·hd^-1^								
Initial to reimplant	1.39	1.45	1.81	2.08	0.042	0.04	0.12	0.18
Reimplant to slaughter	1.26	1.49	1.55	1.79	0.053	<0.01	<0.01	0.91
Overall	1.33^d^	1.48^c^	1.66^b^	1.92^a^	0.036	<0.01	<0.01	0.04
DMI^4^								
Individual, kg/d	8.42^d^	9.18^c^	9.64^b^	10.9^a^	0.197	<0.01	<0.01	0.05
Total, kg/steer	2497	2769	1929	2213	48.8	<0.01	<0.01	0.87
Feed efficiency^5^	0.16	0.16	0.17	0.18	0.001	<0.01	<0.01	0.94
Feed cost, $/steer	1,000.19	1,109.44	772.92	886.62	20.379	<0.01	<0.01	0.87
Feedlot COG, $/kg BW	2.53	2.49	2.33	2.29	0.020	<0.01	<0.01	0.84
Total COG,^6^ $/kg BW	2.53^a^	2.49^b^	2.11^d^	2.24^c^	0.018	<0.01	0.38	<0.01
Total Cost,^7^ $/steer	2,400.58^b^	2,479.43^a^	2,348.34^c^	2,503.63^a^	25.776	0.43	<0.01	0.03
Total net return,^8^ $/steer	352.64^b^	370.78^b^	486.66^a^	374.65^b^	24.189	<0.01	0.01	<0.01

^1^Treatments consisted of calf-fed native beef (NBCF), calf-fed beef × dairy (DBCF), yearling-fed native beef (NBYF), or yearling-fed beef × dairy (DBYF) finishing systems.

^2^BW = body weight; DOF = days on feed; ADG = average daily gain; DMI = dry matter intake; COG = cost of gain.

^3^Standard error of the mean. .

^4^Calculated according to Guiroy et al. (2001) and Tedeschi et al. (2004).

^5^Calculated as kg of BW gain per kg of feed (DM basis).

^6^Calculated as total system cost divided by total gain from stocker and finishing periods.

^7^Calculated as opportunity costs, feed costs, and vet costs.

^8^Calculated as carcass value minus total system cost.

^a, b, c, d^Within a row, interaction means with different subscripts differ at *P* < 0.05.

Average daily gain from feedlot entry to reimplant did not have an interaction (*P* = 0.18) or breed-type effect (*P* = 0.12), but there was a system effect (*P* = 0.04) with the YF system having greater ADG. From reimplant to harvest slaughter, there was no interaction (*P* = 0.91) for ADG, but there were system (*P* ≤ 0.01) and breed type (*P* ≤ 0.01) effects with DB calves and YF system having greater ADG. There was an interactive effect (*P* = 0.04) on overall finishing ADG where DBYF had the greatest ADG followed by NBYF, DBCF, and NBCF, respectively.

In the current experiment for the YF system, the DB calves gained 28% less BW during grazing at MKRRS. But while they were 21 kg lighter at feedlot entry, ADG during the initial finishing period from initial feedlot processing to reimplant was 0.27 kg/d greater for DB than NB which resulted in no difference in BW at reimplant between NBYF and DBYF steers. Compensatory growth is a physiological response to feed restrictions, and a compensatory index can be used to quantify the extent of compensatory growth (Hornick et al., 2000). Compensatory growth index is typically between 50 and 80%, and a compilation of studies in Nebraska reported a range of compensatory growth of grazing calves from 18.7 to 88% (Klopfenstein et al., 1999). However, in the current study, DBYF steers had a compensatory growth index of 114% during the first 87 d at the feedlot leading to no difference in BW at reimplant between the YF systems. The DBYF steers having a 114% compensatory gain index not only indicates there was full compensation or recovery in BW (Hornick et al., 2000) but also additional gain occurred during the first 87 d in the feedlot.

#### Modeled feed intake and feed efficiency.

The model developed for DMI by Guiroy et al. (2001) accounted for 74% of the variation of observed DMI and had no bias. When further evaluated in data from a commercial feedlot, the observed total DM consumption was predicted with a bias of < 1% (Guiroy et al., 2001). The CV observed from Guiroy et al. (2001) was reduced from 8.18% to 3.76% when predicting DMI for groups of 5 calves instead of individuals and was < 2% in groups of 20 calves; therefore, we concluded these calculations could be used to determine DM required for gain in the current research, where group sizes were greater than 20 calves. When looking at model derived daily DMI (Table 4), there was a finishing system × breed type interaction (*P* = 0.05) with DBYF having the greatest DMI followed by NBYF, DBCF, and NBCF, respectively. However, the total finishing DMI did not have an interaction (*P* = 0.87) effect. When looking at system and breed-type effects, the total DMI throughout the finishing phase was 278 ± 35 kg less (*P* ≤ 0.01) for NB calves and was 561 ± 35 kg greater (*P* ≤ 0.01) for the CF system. Feed efficiency (G:F) had breed-type (*P* ≤ 0.01) and system (*P* ≤ 0.01) effects where DB were more efficient than NB and the YF system were more efficient than CF.

In contrast to the results of the current study, there is evidence that feed efficiency in calf-fed systems are more efficient compared with yearling-fed systems, even though feedlot ADG is often greater for yearling-fed cattle than calf-fed cattle (Griffin et al., 2007). Hickok et al. (1992) observed cattle in a calf-fed system gained 0.10 kg/d less and required 0.82 kg less feed per kg of gain than yearling cattle, and results from Williamson et al. (2014) agreed with these findings where Angus-sired beef cattle in a yearling system had a greater ADG and were on feed for fewer days than calf-feds. Winterholler et al. (2008) evaluated a native beef production system where calf-feds were placed on feed at a BW of 228 kg for 169 d and yearling-feds were placed on feed at 445 kg BW for 88 d, and yearling-fed calves had a greater ADG and DMI than calf-fed steers. In the current study, YF steers had greater ADG (1.79 vs 1.41 kg/d) and were on feed for fewer days (201 vs 299 d) than CF steers. Hersom et al. (2004) found that when steers achieved a high rate of gain during grazing, DMI as a percentage of BW was reduced in the feedlot compared to those which had a lower rate of gain on pasture, and furthermore, there would be no differences in overall ADG or feed efficiency in the feedlot.

In the current study, daily DMI was 1.30 kg/d greater in DBYF compared with NBYF. Furthermore, feed efficiency did not differ from DBYF and NBYF, but overall ADG was 0.26 kg greater for DBYF than NBYF. When looking at total DMI in the current study, the CF system required 562 kg more than the YF steers, but since there was no interaction, total DMI did not differ between NBYF and DBYF. Furthermore, increasing BW at feedlot entry typically increases DMI, ADG, and HCW, while reducing feed efficiency (Reuter and Beck, 2013). But in the current study, feed efficiency was numerically greater in DBYF calves, who also had greater DMI, ADG, and HCW. These results agree with Klopfenstein et al. (1999) which reported an increase in DMI and ADG but no differences in feed efficiency are typical of compensatory gain.

#### Costs of production and net returns.

Economic costs and returns are presented in Table 4. There was an interaction for total COG including the grazing phase (*P* ≤ 0.01), total costs (*P* ≤ 0.01), and the total net return (*P* ≤ 0.01). When evaluating the COG for the feedlot phase, there were system (*P* ≤ 0.01) and breed-type (*P* ≤ 0.01) effects with NB and CF steers having higher feedlot COG. Total COG was highest in NBCF followed by DBCF, and steers in NBYF had the lowest total COG. Steers in DBYF had intermediate COG at $2.24 per kg of BW followed by DBCF ($2.49/kg BW) and NBCF ($2.53/kg BW), with NBYF steers had the lowest total COG ($2.11/kg BW). Feed costs increased in DB (*P* ≤ 0.01) and CF (*P* ≤ 0.01) calves by $111.47/steer and $225.04/steer, respectively. Total cost was highest (*P* ≤ 0.01) in DB calves regardless of system with DBYF costing $2,503.63/steer and DBCF costing $2,479.43/steer, and NBCF steers were intermediate at $2,400.58/steer. The NBYF steers had the lowest total cost at $2,348.34/steer. The total system net returns ranged from $352.64/steer to $486.66/steer. The greatest net return was NBYF (*P* ≤ 0.01) steers at $486.66, followed by DBYF, DBCF, and NBCF with returns at $374.65, $370.78, and $352.64, respectively. Based on these results, DB steers were economically competitive with NB steers. The NBCF steers had the smallest return which correlates with the increased total cost and decreased gain and efficiency, whereas NBYF had the lowest total cost with increased gain and efficiency.

### Ultrasound Carcass Characteristics: Pre-Slaughter

The pre-slaughter carcass characteristics estimated by ultrasound are presented in Table 5, and due to unforeseen circumstances, pre-slaughter ultrasounds were not conducted on NBCF steers and thus are not included in the results. At initial processing at the feedyard, REA was greater (*P* ≤ 0.01) for NBYF and DBCF. However, at reimplant, the REA of DBYF was 6.6 ± 1.7 and 16.5 ± 1.5 cm^2^ greater (*P* ≤ 0.01) than NBYF and DBCF, respectively. Also, BFT at initial processing at the feedyard was greatest (*P* ≤ 0.01) in NBYF at 0.48 ± 0.01 cm with DBCF being intermediate (*P* ≤ 0.01) at 0.44 ± 0.01 cm and DBYF being the lowest (*P* ≤ 0.01) at 0.38 ± 0.01 cm. At reimplant, BFT followed reimplant REA where DBYF had the greatest (*P* ≤ 0.01) BFT at 0.91 ± 0.02 cm, and DBCF had the lowest (*P* ≤ 0.01) BFT at 0.66 ± 0.02 with NBYF being intermediate (*P* ≤ 0.01) at 0.81 ± 0.02 cm.

**Table 5.** Effect of calf-fed or yearling-fed finishing systems on ultrasound carcass characteristics preslaughter and body composition of gain in beef × dairy crossbred steers compared to native beef steers.

	Treatments^1^					Effect *P-*values		
Item^2^	NBCF	DBCF	NBYF	DBYF	SEM^3^	System	Breed-type	System × Breed-type
REA, cm^2^								
Initial	-	51.9^a^	54.1^a^	45.1^b^	1.14	<0.01	<0.01	<0.01
Reimplant	-	62.7^c^	73.6^b^	79.2^a^	1.56	<0.01	<0.01	<0.01
Backfat, cm								
Initial	-	0.44^b^	0.48^a^	0.38^c^	0.017	<0.01	<0.01	<0.01
Reimplant	-	0.66^c^	0.81^b^	0.91^a^	0.029	<0.01	<0.01	<0.01
Shrunk body composition^4^								
AFSBW, kg	610	627	630	657	7.3	<0.01	<0.01	0.37
SWG, kg/d	1.52	1.51	1.61	1.64	0.035	<0.01	0.67	0.41
Empty body composition^4^								
BW, kg	598	610	612	615	7.4	0.08	0.15	0.39
BW gain, kg/d	1.46	1.45	1.54	1.57	0.034	<0.01	0.67	0.41
Fat, %	31.8^a^	31.6^a^	31.4^a^	30.1^b^	0.41	<0.01	<0.01	0.04
DOF to 28% EBF	258	247	166	167	4.8	<0.01	0.14	0.11

^1^Treatments consisted of calf-fed native beef (NBCF), calf-fed beef × dairy (DBCF), yearling-fed native beef (NBYF), or yearling-fed beef × dairy (DBYF) finishing systems.

^2^REA = ribeye area; QG = quality grade; AFSBW = final shrunk BW adjusted to common empty body composition of fat (EBF); SWG = shrunk weight gain.

^3^Standard error of the mean. .

^4^Calculated according to Guiroy et al. (2001), and Tedeschi et al. (2004).

^a, b, c^Within a row, interaction means with different subscripts differ at *P* < 0.05.

### Body Composition of Gain

When evaluating body composition of gain (Table 5), shrunk body composition did not have a breed-type × system interaction (*P* ≥ 0.37). However, AFSBW was 22 ± 5 kg heavier (*P* ≤ 0.01) in DB calves and 25 ± 5 kg heavier (*P* ≤ 0.01) in the YF system. Yearling-fed calves had heavier (*P* ≤ 0.01) SWG compared to CF calves. Empty body composition of gain had an interaction effect (*P* = 0.04) for EBF, but were no interaction or breed-type effects (*P* ≥ 0.15) on EBW or EWG. Calves in DBYF had the least (*P* ≤ 0.01) EBF compared to other breed-type by system combinations, but NBCF, NBYF, and DBCF did not differ (*P* ≥ 0.47) from one another. The YF system tended to have a greater (*P* = 0.08) EBW and had a greater (*P* ≤ 0.01) EWG than the CF system. When DOF was calculated to reach 28% EBF, YF calves were calculated to be on feed for 87 ± 3 d fewer (*P* ≤ 0.01) than CF calves. There was no interaction or breed-type effect (*P* ≥ 0.11) for DOF to reach 28% EBF.

Hersom et al. (2004) detected no differences in final EBW in steers grazing winter wheat at different targeted rates of gain or grazing dormant native range before finishing at the targeted BW of 500 kg, but animals targeted for a high pre-finishing rate of gain had the least EBF of 26.9%. In the current study, both EBF and EBW are greater than results reported in Hersom et al. (2004). Steers receiving varying levels of loose DDG during the grazing period in Smith et al. (2021) had an EBF of 30%, similar to EBF of YF steers in the current experiment at 30.8%. When growth is not limited by energy, the proportion of empty body protein is typically decreased and the portion of EBF is typically increased (Tedeschi and Fox, 2020). This is likely what occurred in the NBYF steers as they had a higher EBF and greater BFT at initial finishing processing at the feedlot (Table 5), but DBYF likely had less fat deposition than NBYF during the grazing phase due to their lower gains. Furthermore, fat deposition likely did not recover in the finishing phase since DBYF had a lower EBF compared to other treatments, in contrast to Drouillard and Kuhl (1999). However, DOF to reach 28% of EBF was the same for YF steers, and DOF to reach 28% EBF was greater in CF steers which was expected since YF steers entered the feedlot heavier.

### Carcass Characteristics

Carcass characteristics are presented in Table 6. When evaluating HCW, there was no interaction or breed-type (*P* ≥ 0.15) effect, but YF calves tended (*P* = 0.08) to have 7 ± 4 kg greater HCW. There were also no interactive effects (*P* = 0.33) on USDA QG however, there were breed-type and system (*P* ≤ 0.01) effects with DB and CF system having greater average QG. Dressing percentage was greater in DB (*P* ≤ 0.01) and the CF (*P* ≤ 0.01) system, and there was no breed-type × system interaction (*P* = 0.92). Yield grade exhibited a tendency (*P* = 0.07) for a breed-type × system interaction. When evaluating the tendency for YG, NBCF did not differ (*P* ≥ 0.11) from NBYF and DBCF while DBYF was lower (*P* ≤ 0.02) than NBCF, NBYF, and DBCF, but DBCF was greater (*P* = 0.04) than NBYF. There was no interaction (*P* = 0.38) for marbling score, but there were breed-type and system effects which exhibited greater marbling in DB (*P* ≤ 0.01) calves and the CF (*P* = 0.05) system. Ribeye area did not have an interaction or differ between breed-types (*P* ≥ 0.18), but REA tended (*P* = 0.08) to be greater in YF calves. Backfat thickness had a breed type × finishing system interaction (*P* = 0.03), where the BFT of NBCF calves did not differ (*P* = 0.47) from NBYF calves, but was greater (*P* ≤ 0.03) compared to both DB systems, and DBYF calves had the lowest (*P* ≤ 0.01) BFT. The NBYF calves BFT did not differ (*P* = 0.17) from the DBCF calves and was 0.37 ± 0.07 cm greater (*P* ≤ 0.01) than DBYF calves. Carcass value did not have an interaction or system (*P* ≥ 0.12) effect, but there was a tendency (*P* = 0.08) for DB to have a higher carcass value at $2,864/steer compared to NB at $2,794/steer.

**Table 6.** Effect of calf-fed or yearling-fed finishing systems on carcass characteristics in beef × dairy crossbred steers compared to native beef steers.

	Treatments^1^					Effect *P-*values		
Item^2^	NBCF	DBCF	NBYF	DBYF	SEM^3^	System	Breed-type	System × Breed-type
HCW, kg	430	439	440	443	5.6	0.08	0.15	0.39
DP, % of BW	64.3	61.9	63.7	60.9	0.01	<0.01	<0.01	0.92
REA, cm^2^	97.7	99.3	99.6	99.9	0.95	0.08	0.18	0.33
Backfat, cm	1.70^a^	1.55^b^	1.64^ab^	1.28^c^	0.069	<0.01	<0.01	0.03
YG	3.0	3.0	2.8	2.5	0.13	<0.01	0.12	0.07
Marbling^4^	484	531	473	503	14.3	0.05	<0.01	0.38
QG^5^	5.35	5.83	5.18	5.46	0.143	0.01	<0.01	0.33
Select, %	21.8	5.32	15.5	9.86	-	0.74	<0.01	0.15
Choice, %	76.9	87.2	83.1	83.1	-	0.92	0.24	0.24
Prime, %	1.28	5.32	1.40	1.41	-	0.48	0.42	0.42
Carcass Value, $/steer	2753.22	2850.21	2835.00	2878.28	36.609	0.12	0.08	0.71

^1^Treatments consisted of calf-fed native beef (NBCF), calf-fed beef × dairy (DBCF), yearling-fed native beef (NBYF), or yearling-fed beef × dairy (DBYF) finishing systems.

^2^HCW = hot carcass weight; DP = dressing percentage; REA = ribeye area; YG = yield grade; QG = quality grade.

^3^Standard error of the mean. .

^4^Marbling scores: 200 = Standard+, 300 = Select+, 400 = Choice−, 500 = Choice°, 600 = Choice+, 700 = Prime−, 800 = Prime°, 900 = Prime + (USDA-AMS, 2017).

^5^Quality grades were based on 3 = USDA Standard+, 4 = USDA Select+, 5 = USDA Choice−, 6 = USDA Choice°, 7 = USDA.

Choice+, 8 = USDA Prime−, 9 = USDA Prime°, 10 = USDA Prime+.

^a, b, c, d^Within a row, interaction means with different subscripts differ at *P* < 0.05.

Native beef animals typically have heavier carcasses and more desirable carcass conformation compared to dairy × beef animals (Twomey et al., 2020); however, when straight-bred dairy, dairy × beef, and native beef animals were harvested at a constant HCW, dairy × beef animals had an intermediate REA compared to straight-bred dairy and native beef, and dairy × beef animals had a substantially improved DP (63%) from straight-bred dairy (61%) animals and was similar to native beef (63.5%) cattle (Foraker et al., 2022). Furthermore, Foraker et al. (2022) stated that typical dairy × beef animals should exhibit a DP of 65.4%, REA of 115 cm^2^, USDA YG of 2.4, and USDA Choice QG. However, in the current study, DB steers from both systems had a DP closer to that associated with straight-bred dairy and smaller REA, and DBCF steers had a higher YG than DBYF. Beef × dairy steers did have a greater proportion of carcasses grade USDA Choice which aligns with Foraker et al. (2022). Winterholler et al. (2008) had substantially higher marbling scores than our study’s native beef YF and CF steers (580 vs 479), but native beef steers in both systems had higher REA (83 cm^2^ vs 99 cm^2^) and BFT (1.43 cm vs 1.77 cm). The DB steers in the current experiment had slightly lower marbling score (580 vs 517) and DP (63.4% vs 61.4%) than calves in Winterholler et al. (2008). Carcasses grade USDA Choice (83% vs 85%) and BFT (1.42 cm vs 1.43 cm) were similar between the studies with the current calves having larger REA (83 cm^2^ vs 99 cm^2^). While DB steers did not meet typical carcass characteristics except for USDA QG, there was still no difference in carcass value and returns were intermediate compared to their NB counterparts.

Grote et al. (2025) compared yearling to calf fed finishing systems for beef  ×  dairy crossbred steers. Beef  ×  dairy crossbred steers that had undergone a stocker period grazing high quality pastures sufficient for ADG of 0.92 kg/d, and those calves had 15% greater finishing ADG with larger daily DM intake and lower feed efficiency than calves that were placed directly on feed after weaning at 99 d of age (Grote et al., 2025). Grote et al. (2025) also reported that yearling-fed beef  ×  dairy crossbred steers had greater HCW, improved marbling score, and larger REA than calf-fed beef  ×  dairy crossbred steers. The increase in marbling score resulted in increased percentage of USDA Prime and high Choice quality grades.

### Liver Abscesses

The incidence and grades of liver abscesses are reported in Table 7. Overall incidence of liver abscesses did not have an interactive (*P* = 0.16) effect, but there were effects of breed-type (*P* ≤ 0.01) and system (*P* = 0.01). Beef × dairy steers had over double the incidence of liver abscesses at 82.5% compared to NB calves at 37.3%. When looking at the system effect, YF steers had an incidence of liver abscesses at 70.3% compared to CF steers at 54.2%. There were no differences (*P* ≥ 0.23) in liver abscesses grading A- and A + Open. The amount of grade A liver abscesses was greater (*P* ≤ 0.01) for steers in the YF system. There was an interaction (*P* = 0.05) for liver abscess grade A+, where grade A + liver abscesses were greatest in DBYF and DBCF, NBYF being intermediate, and NBCF having the least occurrence. Beef × dairy steers had a 33% increase (*P* = 0.01) in the incidence of A + Adhered abscesses compared to NB steers, and furthermore, those steers in YF system had a 10% increase (*P* ≤ 0.01) compared to the CF system. The incidence of A + Adhered-Open liver abscesses increased (*P* = 0.04) by 7% in DB calves compared to NB calves.

**Table 7.** Effect of calf-fed or yearling-fed finishing systems on incidence of liver abscesses in beef × dairy crossbred steers compared to native beef steers.

	Treatments^1^					Effect *P-*values		
Item^2^	NBCF	DBCF	NBYF	DBYF	SEM^3^	System	Breed-type	System × Breed-type
Zero, %	74.4	19.7	49.3	12.8	-	0.01	<0.01	0.16
A-, %	1.3	1.4	1.4	1.4	-	0.96	0.96	0.96
A, %	2.6	1.4	12.7	7.0	-	0.01	0.36	0.98
A+, %	2.5^c^	16.9^a^	14.1^b^	16.9^a^	-	0.05	0.01	0.05
A + Open, %	3.8	4.3	2.8	1.4	-	0.23	0.83	0.45
A + Adhered, %	7.7	38.0	14.1	49.3	-	0.01	<0.01	0.74
A + Adhered-Open, %	7.7	18.3	5.6	11.2	-	0.28	0.04	0.78
Overall Abscessed	25.6	80.3	50.7	87.2	-	0.01	<0.01	0.16

^1^Treatments consisted of calf-fed native beef (NBCF), calf-fed beef × dairy (DBCF), yearling-fed native beef (NBYF), or yearling-fed beef × dairy (DBYF) finishing systems.

^2^Liver abscess scores was manually scored and recorded according to the Elanco Liver Check System.

^3^Standard error of the mean. .

^a, b, c, d^Within a row, interaction means with different subscripts differ at *P* < 0.05.

The prevalence of liver abscesses in native beef steers had increased by 25% from 2008 to 2013 and tripled in Holstein steers during the same period (Reinhardt and Hubbert, 2015). The increase in concern of liver abscesses might be a result of the economic incentive to market cattle at heavier HCW, thus increasing DOF (Broadway et al., 2024). The current study agrees with DB calves have a greater propensity for the development of liver abscesses (Foraker et al., 2022). Typically, liver abscess prevalence ranges from 10 to 20% (Reinhardt and Hubbert, 2015), which agrees with the NBCF system, but NBYF was double this and both DB systems were quadrupled. Typically, severe liver abscesses are associated with lighter HCW (Reinhardt and Hubbert, 2015), but here HCW was higher in DB steers compared to their NB system counterparts. While Checkley et al. (2005) saw a decrease in prevalence of liver abscesses when calves were grown using a forage-based diet, the current study saw an increase in liver abscesses in YF calves.

## CONCLUSIONS

Overall, the results of this commercial scale systems research imply that grazing dairy  ×  beef steers prior to feedlot finishing can improve animal performance and certain carcass characteristics. However, post-weaning management did not affect the prevalence of liver abscess since DB steers had a higher percentage of liver abscess as well as more severe liver abscesses. Furthermore, returns for DB steers were between NBYF and NBCF systems which indicates these dairy  ×  beef crossbred calves can be economically competitive to their NB counterparts. This study also showed DB steers exhibited full compensatory gain of reduced performance during grazing in the feedlot phase. Beef × dairy YF steers had the highest ADG and DMI while exhibiting a feedlot COG which slightly smaller and a slightly larger total COG compared to NBYF whereas DBCF had a higher total COG. Grazing DB and NB calves had less feedlot DOF, leading to lower total feed cost. The only interaction in carcass characteristics was the increase for BFT in NBYF and NBCF compared with DBYF. Hot carcass weight and REA were did not differ across breed-type and system, but DB and YF steers had a lower dressing percentage. Fewer DB carcasses graded USDA Select. Further research is needed to determine the environmental implications of post-weaning management systems for dairy  ×  beef crossbred calves and explain the higher incidence of liver abscesses.
